# Emotional Perception of Music in Children with Unilateral Cochlear Implants

**Published:** 2014-10

**Authors:** Sareh Shirvani, Zahra Jafari, Abdolreza Sheibanizadeh, Masoud Motasaddi Zarandy, Shohre Jalaie

**Affiliations:** 1 *Department of Audiology, School of Rehabilitation, Tehran University of Medical Sciences, Tehran, Iran. *; 2 *Department of Basic Sciences in Rehabilitation, School of Rehabilitation Sciences, Rehabilitation Research Center, Iran University of Medical Sciences, Tehran, Iran.*; 3 *Department of Audiology, School of Rehabilitation Sciences, Iran University of Medical Sciences, Tehran, Iran.*; 4 *Otorhinolaryngology Research Center, AmirAlam Hospital, School of Medicine, Tehran University of Medical Sciences, Tehran, Iran.*; 5 *Department of Physiotherapy, School of Rehabilitation, Tehran University of Medical Sciences, Tehran, Iran.*

**Keywords:** Children, Cochlear Implant, Emotion, Music Perception

## Abstract

**Introduction:**

Cochlear implantation (CI) improves language skills among children with hearing loss. However, children with CIs still fall short of fulfilling some other needs, including musical perception. This is often attributed to the biological, technological, and acoustic limitations of CIs. Emotions play a key role in the understanding and enjoyment of music. The present study aimed to investigate the emotional perception of music in children with bilaterally severe-to-profound hearing loss and unilateral CIs.

**Materials and Methods:**

Twenty-five children with congenital severe-to-profound hearing loss and unilateral CIs and 30 children with normal hearing participated in the study. The children’s emotional perceptions of music, as defined by Peretz (1998), were measured. Children were instructed to indicate happy or sad feelings fostered in them by the music by pointing to pictures of faces showing these emotions.

**Results:**

Children with CI obtained significantly lower scores than children with normal hearing, for both happy and sad items of music as well as in overall test scores (P<0.001). Furthermore, both in CI group (P=0.49) and the control one (P<0.001), the happy items were more often recognized correctly than the sad items.

**Conclusion:**

Hearing-impaired children with CIs had poorer emotional perception of music than their normal peers. Due to the importance of music in the development of language, cognitive and social interaction skills, aural rehabilitation programs for children with CIs should focus particularly on music. Furthermore, it is essential to enhance the quality of musical perception by improving the quality of implant prostheses.

## Introduction

More than three decades have passed since the first cochlear implant (CI) surgery took place and, since then, over 200,000 people have benefitted from this technology worldwide (1,2). CI technology transforms acoustic signals into electrical codes and provides hearing enhancement for patients with severe-to-profound hearing loss. It leads to improvements in hearing skills, particularly linguistic and articulation skills, among children (1,3,4). The growing number of CI users worldwide in recent years is a sign of the success of this technology. Accordingly, the CI has been recognized as one of the most beneficial rehabilitation prostheses, and cochlear implantation is known to be one of the safest medical procedures in the world (1,2).

There is a large body of research related to language and speech abilities in individuals with CIs, including studies that compare these with individuals with normal hearing. Topics studied so far include localization abilities (3,5), environmental sound perception (3), speech comprehension in quiet environments or in the presence of background noise (4), and recognition of the speaker's gender (7,8) in children and adults before and after implantation and in comparison with normal-hearing peers. In recent years, the perception of musical elements as a commonplace stimulus in daily life and as a global language has attracted the attention of researchers in the field of audiology (9-18). Musical elements such as rhythm (9-11), pitch, melody and timbre have all been widely studied in different age groups and across genders (9-16). Moreover, in individuals with hearing loss who use various amplification systems, recent studies have explored how music is perceived and what factors influence this (9-18). The findings show that perception of these elements is weaker among individuals with CIs. Moreover, it was found that individuals with hearing loss were better able to perceive rhythm than other musical elements and gained similar rhythm perception scores to the normal-hearing groups (13,17,18).

The perception of the emotions conveyed by music is a crucial factor in understanding music and making listening to music a pleasurable activity. Emotions such as happiness and sadness play a key role in the enjoyment of music and convey deeper musical meanings and concepts (2,19). 

Among this body of research, Hunter's study (2011), which examined the developmental processes of musical emotional perception and also cross-gender differences, deserves particular mention with the application of the Vieillard music test. Participants aged 5, 8, and 11 years and adult subjects were instructed to respond by choosing between images, which were indicative of emotions such as happiness, sadness, threat and peacefulness. Results showed that emotional perception scores increased between the ages of 5 and 8 and 8 and 11 years. At the age of 11 years, the results were the same as those of the adults. Gender was significant at 5 and 8 years of age, with girls outperforming boys. At the age of 11 years and among the adults, however, no significant differences were observed (20).

The current literature review demonstrated that, emotional perceptions of music have not been extensively studied in individuals with CIs. Most of the research done in this area has focused on adults and fewer studies have analyzed the music perceptions of children with hearing loss. Only one such study, by Hopyan (2011), assessed 18 children aged between 7 and 13 years with hearing loss who had unilateral CIs. The findings revealed lower levels of emotional perception in children with CIs compared with their normal peers (2).

The present study is the only study in this area to be conducted in Iranian children. The results of this study may contribute to our understanding of the emotional perception of music among children with CIs. By evaluating the emotional perceptions of music in children with hearing loss, we can highlight new ways of planning more effective rehabilitation programs following implantation. Analyses of the strengths and weaknesses of these children in this respect may help improve their quality of life. The results of this research may be beneficial for researchers interested in hearing, music perception, and CI.

## Materials and Methods

**Participants**

The present cross-sectional study was conducted in Tehran, Iran, from June 2013 to September 2013. Participants included 25 children (12 males and 13 females) aged 6–8 years (mean age: 6.94 years, standard deviation [SD]: 0.68 years) who had unilateral CIs. Thirty other children with normal hearing (15 males and 15 females) aged between 6 and 8 years (mean age: 6.84 years, SD: 0.70 years) also participated in the study. All children were monolingual, speaking only Persian, and all were right-handed (21). Children in the CI group were experienced users with an average of 3.3 years (SD=0.7) experience with the CI device, and a mean age of CI activation of 3.6 years (SD=0.7). All participants took a Raven’s 32-item nonverbal intelligence test and those who were found to be of normal intelligence and scored above 85 were included in the study (22). A questionnaire to assess depression was also employed, and children who were depressed were excluded from the study (23). In the control group, otoscopic examination and hearing screening pure tone audiometry were undertaken. Only children with normal peripheral hearing (average hearing thresholds for 0.5, 1 and 2 KHz between 0 and 15 dB HL), took part in the study (24). All participants with hearing loss suffered from severe-to-profound congenital hearing loss, used Nucleus prostheses (CI24RE) and the ACE processing strategy on the right side, and had at least 2 years of experience. Children with auditory neuropathy disorder, neurological impairment, psychological illnesses, growth disorders, head trauma or previous music training were not included in the study. In order to establish the above criteria, we interviewed the parents and also reviewed the children's medical and CIs files. This study was approved by the Tehran University of Medical Sciences ethics committee.

Procedure

**Fig 1:**
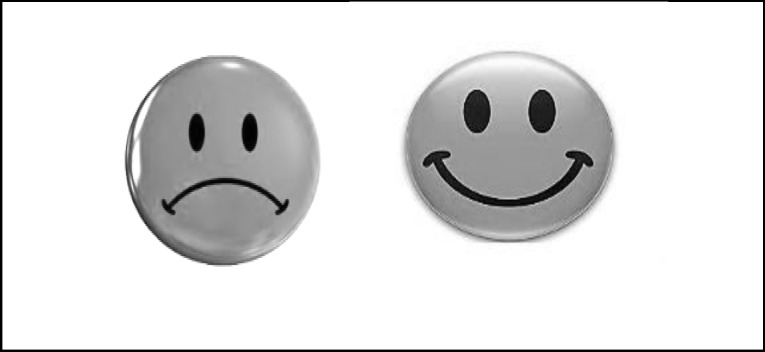
Sad and happy faces were used to responding to test items of emotion perception of music

Tests were conducted in a quiet room with minimal visual distractions. The child was seated on a comfortable chair facing a loud speaker at zero degree azimuth at a distance of 1 m. This was the same set-up as in the previous studies that investigated music perception in clinical pediatric patient populations (2). The most comfortable level for each child was determined by playing a music track that was different to the ones used in the actual test, asking questions about the child's comfort level and changing the volume accordingly. This level was kept constant throughout the study. The Peretz test was employed in order to investigate the musical perceptual ability of the child (19). The test was designed in 1998 with the aim of providing a sufficiently complex and meaningful structure that could be used instead of a simple sequence of tones and includes 32 musical items. These musical tracks consist of four styles: Baroque (Bach, Albinoni), Classical (Mozart), Romantic (Verdi), and Contemporary (Ravel). Half of these items evoked a sense of happiness, whereas the other half evoked a sense of sadness. Items lasted between 7 and 33 s (mean: 15.8 s), and the test score for each item was determined by the percentage of correct answers (happy/sad) (19). After training the child in how to carry out the test, the items were played at a constant intensity at the most comfortable level. The most comfortable level was approximately 35 dB HL for the control group and 75 dB HL for the CI group. Musical items were broadcast through a loud speaker connected to a portable computer. There was no noticeable distortion of the sound field loudspeaker at high intensity levels. After listening to each track, the child would look at two pictures of faces, one smiling and the other sad (as shown in Fig. 1) and was asked to point to one of them. Each time, participants were given 15 s to choose the correct emotion. In cases where the child was unable to answer, the track was played once again (with each track being played a maximum of two times). No reinforcement or hints were allowed during the test.

The SPSS package (version 18) was used to analyze the data, with p-values of ≤0.05 considered to be statistically significant. A Kolmogorov–Smirnov test was performed to assess the normality of the data. To compare the between-group means of musical emotional perception (both in total and separately for happy and sad items) in the two groups, a Mann–Whitney test and independent sample t-test were employed. A paired sample t-test (for the CI group) and Wilcoxon–Signed–Rank test (for the control group) was used to make within-group comparisons between happy and sad items.

## Results

Data analysis revealed statically significant differences between children with unilateral CIs and children with normal hearing in overall test score (P<0.001) and in the individual happy (P<0.001) and sad items (P<0.001). Table 1 shows the mean and SD of the test scores for happy and sad items, along with the total test scores for each group. P-values are also indicated.

Furthermore, both in the CI group (P=0.49) and the normal-hearing group (P< 0.001), happy items were recognized more correctly than sad ones.

**Table 1:** Mean scores of music emotional perception in two groups; for happy, sad items and overall

Music emotional perception test scores		Children with normal hearing (n=30)	Children with unilateral CI (n=25)	P value
Sad score (%)	Mean	87.28	55.91	>0.001
	SD	8.43	9.23	
Happy score (%)	Mean	95.83	57.95	>0.001
	SD	6.21	8.44	
Total score (%)	Mean	91.24	56.20	>0.001
	SD	5.99	6.16	

## Discussion

Music is a global language and a fast and reliable way to experience, understand and convey emotions. It may be viewed as the primary perceptual stimulus of the auditory system. After its auditory system is formed, the embryo first hears its mother's heartbeat, which is purely rhythmic (1). Following birth, the infant first perceives the tones of speech before learning the actual sounds of its native language. This indicates the importance of processing and understanding music and its elements in human development (25). On the other hand, a body of research using imaging techniques has indicated the existence of adjacent or even similar brain areas that are stimulated by speech and music. These are the anterior and posterior area of the superior temporal gyrus, superior temporal sulcus, and supra marginal gyrus (26-29). Therefore, a strong perception of music can facilitate the perception and processing of speech and language (30-32). This issue is of great importance in children with hearing loss; i.e. children who were unable to perceive correct speech in their critical period of language development due to hearing loss (32). Unfortunately, in some places these children are often given a CI after this critical period has passed. Once they enter rehabilitation programs having received a prosthesis, attempts are made to compensate for these deficiencies. In this period, music can be used to stimulate those areas in the brain that are effective in perceiving speech and language. This will considerably expedite this process and, to some extent, reduces the access time to the target skills (32,33(. Due to the plasticity and flexibility of the nervous system, the possibility of acquiring particular skills, and the enhanced brain adaptability at younger ages, music can be used in post-implant rehabilitation programs as a stimulant, which is capable of altering the functionality of brain. This can, in turn, influence the flexibility of the nervous system (32-36). 

In terms of musical perception among people with hearing loss, emotional perception is the theme which is of the most interest in the present study. In the present study, Peretz test scores (happiness, sadness, and total score) were significantly lower in children with unilateral CIs than those of normal hearing. This is indicative of the lowered ability of children with hearing loss in accurately perceiving emotions in music. These results are in line with those of Hopyan's study (2). In Hopyan's study, which had 18 participants with unilateral CIs, including 11 girls and 7 boys, all aged 7–13 years (average age: 10.2 yrs), the hearing loss group obtained lower scores. A better perception of happiness than sadness was reported in both groups. The Peretz test of musical perception was also used in Hopyan's study (2). Hopyan reported that age at CI activation and time since CI activation were both uncorrelated with outcome measures (2).

An individual with normal hearing uses spectral and temporal cues to perceive musical elements (19). An individual with hearing loss, however, faces difficulty in perceiving these cues in their entirety due to the limitations of the hearing system, such as the number and patterns of the remaining cells of the spiral ganglion (18,37). The processing strategies designed for CIs are focused particularly on the transmission of temporal cues and, despite the aim of improving speech, this fails to provide sufficient information to understand pitch-related information (37,38). Therefore, in people with CIs, temporal cues are transmitted well, but spectral cues are dependent upon a correct understanding of frequencies and the pitch is not appropriately conveyed. This tends to lead to poor perception of music (18,19). Furthermore, the average depth of insertion of electrodes into the cochlea is 20 mm, while the human cochlea is 33 mm in length. Therefore, through this electrode insertion, the transmittable frequency field is between 200 and 8500 Hz. This is sufficient to comprehend speech; however, the comprehension of music requires more low frequency information to be transmitted (36). As a result, a proper perception of pitch and emotions is not possible. Concerning the biological, structural, and acoustic limitations that face CI users, high-quality music similar to that perceived by people with normal hearing will not be provided for CI patients (37). However, it is necessary to examine closely the precise perception of the different aspects of music as a stimulus (e.g., pitch, timbre, and melody). It is also necessary to understand every difficulty that children with hearing loss encounter when trying to perceive emotions through music. After compensating for these difficulties, children with hearing loss can better perceive musical emotions. This not only makes music more enjoyable for these children, but it would also optimize cochlear implantation as a therapeutic option, resulting in a better and improved message reception and bidirectional and efficient communication (2,19).

In the present study, the average score of musical emotional perception in children with CIs (mean=56.20%, SD=6.16%) was significantly lower than that found in Hopyan's study (mean=77.5%, SD=12.7%), while the average score in the two study groups with normal hearing was very close together (present study: average score=91.24%, SD=5.99, Hopyan's study: average score=97.3%, SD=2.7). This may be due to the higher mean age of the participants in Hopyan's study (4 years of difference), and a better auditory experience and increased probability of having been exposed to music (2). Similar research in different age groups may provide more information about the impact of age on musical emotional perception. Additionally, lower scores in hearing-impaired children can be potentially due to the lack of rehabilitation programs in the use of music stimuli. Also one key factor in musical perception, particularly among people with CIs, is the variety of processing strategies used in the CI which can lead to different results. Among the most important processing strategies are Advanced Combination Encoders (ACE), Continuous Interleaved Sampling (CIS), multipeak speech coding strategy (MPEAK), spectral-peak speech coding strategy (SPEAK), MP3000, FSP, SAS, and MPS (39). In the present study, all participants used the ACE processing strategy, but the processing strategy of the Hopyan study was not mentioned (2). Future research comparing the effects of different processing strategies upon musical perception could pave the way for the design and implementation of more successful strategies. Further research should focus on the examination of instructional interventions and practice to improve musical emotional perception.

Another important finding of this study is that feelings of happiness were more likely to be perceived correctly than those of sadness in both normal and hearing-impaired groups, which in the case of children with hearing loss is similar to Hopyan's results. However, in the present study, this difference was more significant in the group with normal hearing, while in the Hopyan study, in children with normal hearing, significant differences in the perception of happiness and sadness was not reported (2). 

Two influential factors in comprehending happiness and sadness in music are tempo and mode; of which the former plays the larger role. A slow tempo or few beats per minute evoke sad moods, while a fast tempo and more beats per minute tend to evoke happy moods. Mode, which is related to the subset of pitches selected in a musical track, evokes sadness in its minor state and happiness in its major state (19). At the age of five, children can distinguish between happiness and sadness through tempo-related information. A more precise recognition is actualized at the age of six years through the understanding and perception of mode. A six-year-old child can easily differentiate between happy and sad music, based solely on its mode and tempo. This ability is maintained throughout the course of life (40). This information can be transmitted in less than half a second. The time required to transmit this information is shorter for happy music than for sad music. Happy feelings related to music are therefore conveyed faster and more efficiently than sad ones. This discrepancy is more evident in people with normal hearing than among those with hearing loss, since these people have difficulties relating to information transmission (19,39). People with hearing loss can, by relying solely on their perception of rhythmic information (which is similar to that of normal-hearing people), distinguish between happy and sad tracks above the level that would be expected through chance alone. In this case, happy tracks, which tend to have a stronger rhythm, were more easily identified than sad ones (38).

In the present study, all children used a nucleus prostheses (CI24RE) with the ACE processing strategy in the right ear. It is possible that research into other known CI prostheses and processing strategies, and in particular, a comparison of the emotional perception of music between right ear and left ear cochlear implantations, could provide more interesting results in this regards. 

## Conclusion

The score for emotional perception of music in this study was significantly lower in the implant group than in the normal-hearing group. Moreover, the average score obtained for happy items was higher than that for sad ones. Failure to perceive the emotions conveyed within music may negatively affect the quality of life and social relationships of children with CIs. This could also further deprive these children of the joy that music can bring, and of the proven positive effects that music can have on language and speaking skills. In light of the present findings, we suggest that following cochlear implantation, rehabilitation programs place greater emphasize on the use of music as an effective tool for teaching speech comprehension and language skills. Children, particularly those in younger age groups who still have high levels of neural plasticity and adaptability, need to be exposed to this powerful, efficient and joyful stimulus. Processing a wider range of frequencies through the use of hearing aids in the opposite ear (bimodal fitting) maybe create a better perception of musical emotions by increasing the quality of transmitted spectral information. It is suggested that this line of research should be pursued in the future.
